# Combined endodontic and periodontal management of a class 3 invasive cervical resorption in a mandibular first molar

**DOI:** 10.1002/ccr3.1785

**Published:** 2018-09-04

**Authors:** Takayoshi Nagahara, Katsuhiro Takeda, Yusuke Aida, Tomoyuki Iwata, Ryoichi Yagi, Hidemi Kurihara, Hideki Shiba

**Affiliations:** ^1^ Nippon Kokan Fukuyama Hospital Hiroshima Japan; ^2^ Department of Periodontal Medicine Graduate School of Biomedical and Health Sciences Hiroshima University Hiroshima Japan; ^3^ Department of Biological Endodontics Graduate School of Biomedical and Health Sciences Hiroshima University Hiroshima Japan

**Keywords:** cone‐beam computed tomography, free gingival graft, invasive cervical resorption, mineral trioxide aggregate, perforation repair

## Abstract

Dental radiography and cone‐beam computed tomography revealed the left mandibular first molar in a 68‐year‐old female patient with Heithersay Class 3 invasive cervical resorption (ICR). The inhibition of ICR progression and environmental improvement in and around the affected tooth through combined endodontic and periodontal treatments led to a favorable clinical outcome.

## 1 INTRODUCTION

Invasive cervical resorption (ICR) is a relatively uncommon form of external resorption. This externally resorptive process is characterized by a cervical location and leads to progressive and destructive loss of tooth structure. The etiology of ICR is unclear, however, mechanical, inflammatory, autoimmune, or infectious stimulus are considered predisposing factors of ICR.^1^, ^2^, ^3^, ^4^, ^5^, ^6^, ^7^ The extent of the resorptive defect inside the tooth can be used to classify lesions from Class 1‐4 according to the radiographic appearance of the process.^1^, ^3^, ^4^, ^6^, ^7^ Treatment is generally successful in Class 1 and 2 cases, reasonably successful in Class 3 cases and generally unsuccessful in Class 4 cases.^2^

The cases of anterior teeth outnumber those of posterior teeth in the treatment of ICR.^8^ ICR aggressively erodes cervical area of a tooth. Since a molar, in general, has plural tooth roots and the furcation area, the complexity of the resorption state in the molar from the anatomical view point may make the treatment of the ICR difficult. This report describes combined endodontic and periodontal approaches that led to a favorable outcome for the left mandibular first molar which was diagnosed as Heithersay Class 3 ICR.

## 2 CASE REPORT

**Figure 1:**
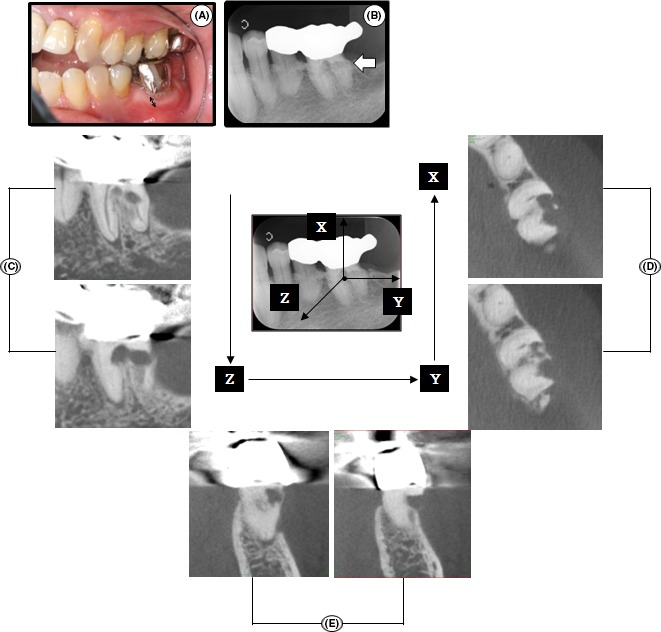
Intraoral photograph, dental radiograph, and CBCT images of tooth 36 at the first visit. A, Clinical view of left maxillary and mandibular quadrants. Attached gingiva was observed around tooth 36 (double‐headed arrow). B, Dental radiograph. An “irregular mottled” or “moth‐eaten” (irregular shape) appearance was observed (white arrow). C‐E, A series of CBCT images. Sagittal (C), axial (D), and coronal slices (E) clearly showed the resorptive lesion with an “outside‐in” appearance

A 68‐year‐old female patient visited Hiroshima University Hospital with a chief complaint of gingival discomfort around the left mandibular first molar (tooth 36). She had no history of trauma, orthodontic treatment, or bleaching, however, the affected tooth and the second premolar were abutments of a three‐unit metal cantilever bridge. The pontic, which was connected to the two crowns, extended into the missing second molar space. This bridge had been fixed approximately 15 years before the first visit. She had a malocclusion, open bite, and crossbite (Figure 1A). There was no relevant medical history. There was bleeding on probing on 36 with a pocket depth of 3 mm in all areas except the buccal furcation (6 mm). The tooth responded positively to thermal and electric pulp vitality tests by PULPER® (GC Dental Industrial Corp.) and Digitest® (Parkell) after removal of the metal crown. Attached gingiva was observed around tooth 36 (Figure 1A). There was no spontaneous and percussion pain. A dental radiograph showed a radiolucent lesion extending from the distocervical level to the coronal third of the root and no pathological change around the root apex (Figure 1B). A radiographic examination revealed an “irregular mottled” or “moth‐eaten” pattern in the main lesion area of the cervical area and the lesion showed a radiopaque mineralized outline of the canal through radiolucency of the external resorptive defect (Figure 1B).^3^, ^5^, ^7^ To determine the extent and depth of the lesion area in three spatial levels, cone‐beam computed tomography (CBCT) was performed. In sagittal and axial slices, we observed the entry points of the granulomatous tissue, which were located in the distal and furcation areas of the buccal cervical root (Figure 1C,D). Communication with the root canal was observed in sagittal and axial slices (Figure 1C,D). Buccal alveolar bone resorption, which continued with ICR, was observed in coronal slices (Figure 1E). A series of CBCT images showed the resorptive lesion with an “outside‐in” appearance.^7^ According to dental radiography and CBCT findings, tooth 36 was diagnosed as Heithersay Class 3 ICR. In addition, the new three‐dimensional classification of ICR using CBCT showed that the ICR in this case was classified as 3Bp (ICR lesion height 3: extends into the mid‐third of the root, circumferential spread B: <180°, proximity to the root canal p: probable pulpal involvement).^9^

Debridement, perforation repair of the resorptive area with mineral trioxide aggregate (MTA) (ProRoot MTA®, Dentsply Maillefer) and root canal treatment were performed with the patient's informed consent.

**Figure 2:**
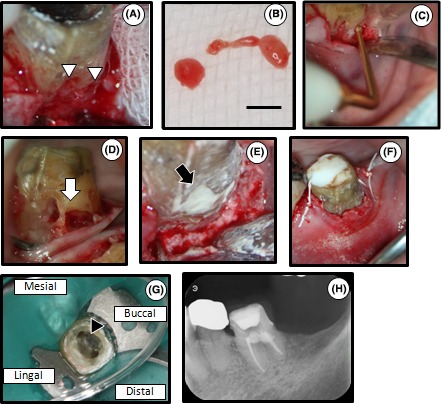
The first stage of treatment: removal of the granulomatous tissue, endodontic treatment and MTA filling of the defect. A, The surgical site. The cervical resorptive site was housed with the granulomatous tissue (white arrowheads). B, A part of the granulomatous tissue removed by the spoon excavator. Scale bar=3 mm. C, Complete removal of the granulomatous tissue by sonic instrument. D, Intraoral photograph after the removal of the granulomatous tissue. Pulp exposure measuring approximately 3 mm in diameter was observed at the resorptive site (white arrow). E, Filling of the resorptive site with MTA (black arrow). F, Tooth 36 after suturing. G, Location of root canal orifices and hardened MTA. Hardened MTA was observed (black arrowhead). The patient exhibited no clinical symptoms. H, Dental radiograph after root canal filling

The full thickness flap was raised to allow visualization of the entry point of the granulomatous (Figure 2A), which was subsequently removed from the surgical site with a spoon excavator (Figure 2B). A sonic instrument (Varios 750®; Nakanishi Inc.) was then used to remove the residual granulomatous tissue (Figure 2C).^10^ As a result, a larger defect size was observed and the pulp was exposed. Because the exposed size measured approximately 3 mm in diameter (Figure 2D), endodontic treatment was performed. The working length was determined by using an electric apex locator (Root ZX®; J Morita). The root canals were cleaned and shaped by a rotary NiTi file (size 45/.04, K3®, SybronEndo) using the crown‐down technique. MTA was subsequently used for perforation and defect repair (Figure 2E). The cavity was temporarily double‐sealed with temporary stopping (Temporary stopping®, GC Dental Industrial Corp.) and glass ionomer cement (Base cement®, Shofu Inc.). The flap was then repositioned without tension and sutured interproximally (Figure 2F).

The patient was recalled 1 week after the operation. The tooth had been asymptomatic. The tooth was isolated with a rubber dam. After removal of the temporary seal (Figure 2G), the root canals were copiously irrigated with sodium hypochlorite (Neo Cleaner®, Neo Dental) and ethylenediaminetetraacetic acid (Smear Clean®, Nippon Shika Yakuhin KK). Calcium hydroxide (Calcipex Plane II®, Nippon Shika Yakuhin KK) was used as an intracanal medication. Since the patient exhibited no clinical symptoms after 3 months, bacterial examination using an anaerobic culture system was performed to evaluate the presence or absence of bacteria in the root canals.^11^ The root canals were filled with gutta‐percha (Dentsply Maillefer) and sealers (Canals‐N®, Showa Yakuhin Kako Co., Ltd.) using the single‐cone technique since the bacterial examination was negative (Figure 2H).

**Figure 3:**
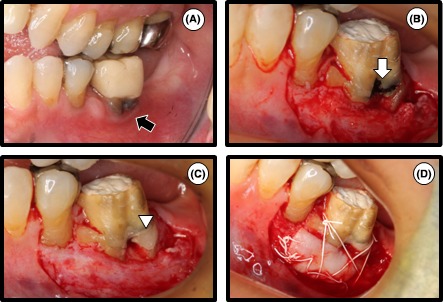
The second stage of treatment: free gingival graft. A, One month after root canal filling. Loss of attached gingiva was observed around tooth 36 with the temporary crown (black arrow). B, Clinical view of the partial‐thickness flap. Hardened MTA was observed (white arrow). C, Covering of the surface of hardened MTA with glass ionomer cement (white arrowhead). D, The graft was sutured at the recipient site to ensure immobilization. Graft tissue was harvested from the palate

One month after root canal filling, thin attached gingiva and plaque accumulation on the cervical contour were observed in tooth 36 with the temporary crown (Figure 3A). The width of attached gingiva after the first stage of treatment (Figure 3A) was narrower than that at the first visit (Figure 1A). To increase the width of attached gingiva surrounding the tooth, free gingival graft (FGG) was performed. The graft recipient site was prepared by partial‐thickness dissection (Figure 3B). Hardened MTA (Figure 3B), which had been used to fill the resorptive site in the first stage of treatment, was covered with glass ionomer cement (Fuji IX®, GC Dental Industrial Corp.) using the sandwich technique^12^ or multidisciplinary approach^13^ (Figure 3C). Donor tissue was procured from the palate. The graft was then sutured at the coronal margin to ensure immobilization (Figure 3D).

**Figure 4:**
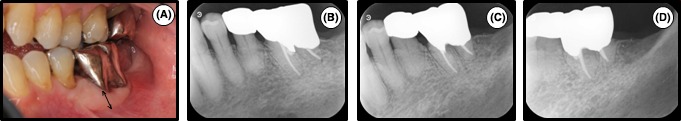
Follow‐up. A, Intraoral photograph at the 3‐year follow‐up. A full metal crown was set on the tooth. Unusual views of the circumference of the gum were not accepted. An adequate width of attached gingiva was observed around tooth 36 (double‐headed arrow). B‐D, Dental radiographs were taken at three different horizontal angles at the 3‐year follow‐up (B, mesio‐eccentric projection; C, orthoradial projection; D, disto‐eccentric projection). Unusual views were not seen

The 3‐year follow‐up demonstrated that the tooth with the full metal crown exhibited no clinical symptoms (eg, no pain, swelling, or mobility with a periodontal pocket depth <3 mm) and adequate width of attached gingival was maintained (Figure 4A) compared with before FGG (Figure 3A). In radiographs, the affected tooth and its surrounding tissues demonstrated no pathological changes (Figure 4B‐D).

## 3 DISCUSSION

In some Heithersay Class 2 and Class 3 cases, the lesion is very close to the pulp and dentin between the lesion and the pulp is very thin. Therefore, endodontic treatment may be necessary since a risk of perforation into the pulp cavity exists upon removing the granulomatous tissues.^6^, ^14^ In fact, the mean width of this thin layer has been reported to be approximately 210 μm.^15^ Histopathologically, the lesions contain fibrovascular tissue with resorbing classic cells adjacent to the dentin surface.^6^ More advanced lesions display fibro‐osseous characteristics with deposition of ectopic bone‐like tissues both within the resorbed tissue and on the dentin surface.^6^ Secondary invasion of microorganisms into the pulp or periodontal ligament space will elicit a inflammatory response.^6^ Removal of the granulomatous tissue in the resorptive area and restoration of the defect after removal are essential as conservative therapy. CBCT can assist in an accurate diagnosis, leading to appropriate treatment.^16^, ^17^, ^18^, ^19^ As CBCT images informed us of the resorptive extent and invasion of the lesion into radicular pulp, this case was diagnosed as Heithersay Class 3 ICR and was conducted surgical treatment for removal of the granulomatous tissue, perforation, and defect repair with MTA and endodontic treatment.

There are several successful case reports using nonsurgical approaches, such as topical application of 90% aqueous solution of trichloroacetic acid (TCA) to the granulomatous tissues, orthodontic extrusion, and low‐power neodymium‐doped yttrium‐aluminum‐garnet (Nd:YAG) laser irradiation.^3^, ^7^, ^20^ TCA is recommended for removal of the granulomatous tissue.^3^ In the present case, the granulomatous tissue in the resorptive area was removed by curettage and a sonic instrument^10^ without TCA application^1^, ^5^, ^21^ in the first stage of treatment. Furthermore, long‐term use of calcium hydroxide was applied to encourage the prevention of resorption.^22^

Amalgam, composite resin, glass ionomer cement, resin‐modified glass ionomer cement, and MTA are available to fill the defect during surgical treatments, such as apically positioned flap and repositioning flap.^3^, ^5^, ^7^, ^21^, ^23^, ^24^, ^25^ MTA has many favorable properties including good sealing ability, biocompatibility, bactericidal effect, radiopacity, and the ability to harden even in the presence of bodily fluids.^26^, ^27^, ^28^ When MTA was applied to seal the perforation, it induced periodontium repair and new cementum formation.^27^, ^29^ Therefore, MTA was used for perforation and defect repair after the removal of the granulomatous tissue at the diseased site. However, the surface of hardened MTA was rough. The previous studies have shown that the interaction between glass ionomer cement and the tooth structure may be similar to that between glass ionomer cement and MTA, suggesting the setting of glass ionomer cement is not hindered by MTA.^30^, ^31^ Since a rough surface on a tooth tends to be a plaque retention factor, the surface was covered with glass ionomer cement to eliminate roughness in this case.

The fundamental objective of endodontic treatment was to eliminate bacteria in the root canal system because they play an important role in the onset and development of periapical lesions.^32^ Therefore, a bacterial examination using an anaerobic culture system is useful for endodontic treatment.^11^, ^32^ In this case report, since the perforation had occurred during the removal of the granulomatous tissue with the gingival flap raised, the rubber dam isolation was not done during the first endodontic treatment, that is, pulpectomy. This indicated the possibility of secondary bacterial contamination through the communication between root canals and oral cavity which would elicit unfavorable outcome. To deny this possibility, the bacterial examination was conducted. The result of the bacteria culture was negative, suggesting that there is no bacterial contamination from the oral cavity in the root canals after endodontic perforation repair with MTA in this case. Since no clinical symptoms were confirmed, the root canal filling was performed.

In this case, since attached gingiva had been lost and, as a result, plaque had readily accumulated at the buccal cervical contour of tooth 36 after the first surgical treatment, FGG was performed at the affected tooth to gain attached gingiva.^33^ Although it has been controversial whether attached gingiva that is keratinized is indispensable to maintain gingival health and prevent gingival inflammation, FGG in this case provided better access for tooth brushing and improved oral hygiene.^34^

## 4 CONCLUSION

The diagnosis of ICR using CBCT, perforation, and defect repair by filling with MTA at the diseased site, endodontic treatment with bacterial examination and a healthier oral environment by FGG contributed to a favorable clinical outcome in this case. Thus, this case report has revealed that combined endodontic and periodontal treatments have value in treating ICR of a molar.

## CONFLICTS OF INTERESTS

None declared for all authors.

## AUTHORSHIP

TN, KT, YA, TI, RY, HK and HS: drafted the manuscript and contributed to treatment of the patient. All authors have read and approved the final manuscript.
